# A Case of Empyema

**Published:** 1888-11

**Authors:** C. A. Brooks

**Affiliations:** Americus, Ga.


### A CASE OF EMPYEMA.

BY C. A. BROOKS, M. D., AMERICUS, GA.

A case of empyema which has recently fallen under my care, presented some points of interest which prompt me to present it to the profession.

On March 28th, I was called to see little A—, the son of Mr. N—, a boy eight years old. Though small to his age, he seemed well nourished and family history good. When about six weeks old he had whooping cough, complicated by bronchitis, from which he suffered for quite a long while and was very delicate throughout his infantile life. After that he seemed to recover entirely, gained flesh and strength and had been quite healthy for several years, with the exception of occasionally, when he contracted the slightest cold.

The evening before I was called to see him, he had slipped and fallen in running over a wet floor. He complained somewhat at the time, but was soon quieted and he ran along to play much as usual for several hours, little importance being attached to it. But in four or five hours night came on and he came in complaining of a distressing pain over the region of the third rib on left side (the side upon which he had fallen). He was put to bed, had some fever and was quite restless throughout the night. When seen on the morning of the 28th, he had a temperature of 103° F., pulse rapid and full, slight cough, severe pain over circumscribed area, respiration rapid and restrained. A physical examination showed a well-developed chest, no broken rib, slight dullness on percussion and rough friction sounds over a circumscribed area in the painful region, but no crepitus. He was given opiates to relieve the pain, five-grain doses of antipyrine at appropriate intervals to control the temperature, straps of adhesive plaster extending from spine to sternum to restrain the movements of respiration on that side (from which he experienced great relief), his bowels moved with an aperient. In the evening found the temperature down to 101°, the little patient quite comfortable. This treatment was continued, as the circumstances seemed to require, for about ten days, after which the febrile symptoms disappeared, the plasters were removed and convalescence seemed to have set in. A physical examination showed at this time a slight accumulation of liquid in the chest, which we thought was serum and hoped under appropriate tonics, etc., would soon be absorbed. He was accordingly placed upon elixir of beef, wine and iron, under which his appetite and digestion improved to a very considerable extent, but there was little improvement in point of flesh and strength, and instead of a diminution of the fluid in chest it was increased.

His appetite was good and digestion still seemed all right, however, and he was placed upon Fellows' syr. of the hypophosphites comp., with still the hope that the effusion might be absorbed. But instead it continued to accumulate, the little patient became dull and listless, hectic fevers set in, and the diagnosis of empyema, which had been feared from the first and strongly suspected for quite a while, was pretty surely made, and finally on the 28th of May we determined to aspirate. There was considerable bulging of the chest over the left posterior surface of the lower part of the chest, and auscultation and percussion indicated that the left side was filled with pus, the heart being displaced. The needle downward and to the right of the aspirator was introduced in the seventh intercostal space in a line with the angle of the scapula, and six and one-half ounces of pus withdrawn. He began at once to improve. He was kept on syr. hypo. comp. and his appetite and digestion improved to such an extent that restraint was necessary; the hectic fevers disappeared, and, notwithstanding the small amount of liquid withdrawn, we flattered ourselves that the trouble was over.

The cough, however, continued to some extent, and later an accumulation of pus at upper and anterior portion of the chest occupying the space between the second and fifth rib, and it had pointed between fourth and fifth. Here on June 6th a free opening was made under antiseptic precautions; there was a discharge of several ounces of pus, after which it was dressed with borated cotton and left open. This continued to discharge and the dressing was changed once a day for fourteen days, when it ceased to discharge pus and soon healed nicely. Immediately upon the discharge of the pus from upper and anterior cavity, symptoms of general dropsy set in. But he was placed upon a prescription of ferri et pot tartrate, syr. scillæ, ext. jaborandi, tr. digitalis, with sherry wine, and in about ten days these symptoms yielded kindly, the color of health returned to his cheeks, his wonted life and spirits were his again and to-day he seems perfectly well with slight retraction of chest. The upper lobe of the lung, I am sure, is entirely restored to its function—all activity—and the lower, though somewhat injured, seems in a fair way to recover.

*Later.*—At this writing, August 21st, I am able to report the little patient entirely well, plump and rosy. The whole lung on the injured side is perfectly restored.

The points of special interest in this case seem to me—

1st. The fact that there were evidently two separate and distinct abscesses or pus cavities formed, probably due to the adhesion of the pleura covering the lung to the chest-wall in the region of the primary circumscribed acute inflammation. This seemed to be the dividing line.

2nd. The fact that a single evacuation of the pus resulted in a complete cure.

3rd. The symptoms of general dropsy following the evacuation of the pus around the heart. This I am inclined to attribute to the sudden removal of the pressure which doubtless caused a certain amount of dilatation of that organ.

Of course I am well aware that this is frequently the result of long-continued suppuration, which causes fatty degeneration of the kidneys and other organs. But the fact of its following immediately upon the withdrawal of the pus from the immediate neighborhood of the heart, and the fact of the complete and prompt recovery taking place so soon after, confirm me in my first opinion that it was due to the embarrassment of the heart's action, which came from the dilation consequent upon the sudden withdrawal of pus which had so long surrounded it.

4th. A point of interest arises in regard to the cause of the acute pleuritis. (a) Was it a broken rib? (b) Could it have been caused by the fall without a broken rib? Or was it idiopathic, caused by the exposure in running over the wet floor in the rain?

(a) I could get no crepitus and could trace all the ribs in the painful region without discovering any breach; he felt no pain for several hours after the fall, and when after ten days the plasters were removed, there was no inconvenience felt and no sign of the deposit of provisional callus which would necessarily have followed such an injury. (b) I am inclined to think a severe concussion consequent upon a fall upon the side, producing more or less contusion, etc., might have caused a circumscribed acute plueritis, or, as I suggested, it might have been idiopathic from the exposure in the rain which he had undergone during the afternoon of his fall.

